# Carbon sequestration rates indicate ecosystem recovery following human disturbance in the equatorial Andes

**DOI:** 10.1371/journal.pone.0230612

**Published:** 2020-03-30

**Authors:** Marco Calderón-Loor, Francisco Cuesta, Esteban Pinto, William D. Gosling

**Affiliations:** 1 Centre for Integrative Ecology, School of Life and Environmental Sciences, Deakin University, Melbourne, Australia; 2 Grupo de Investigación de Biodiversidad, Medio Ambiente y Salud–BIOMAS, Universidad de las Américas (UDLA), Quito, Ecuador; 3 Department of Ecosystem & Landscape Dynamics, Institute for Biodiversity & Ecosystem Dynamics (IBED), University of Amsterdam, Amsterdam, Netherlands; 4 Biodiversity Department, Consorcio para el Desarrollo Sostenible de la Ecorregión Andina (CONDESAN), Quito, Ecuador; University of Maryland, UNITED STATES

## Abstract

Few studies exist that document how high-elevation Andean ecosystems recover naturally after the cessation of human activities and this can limit the implementation of cost-effective restoration actions. We assessed Andean forest (*Polylepis* stands) and páramo grassland recovery along an elevation gradient (3,600–4,350 m.a.s.l.) in the Yanacocha Reserve (Ecuador) where natural recovery has been allowed since 1995. Within the Yanacocha Reserve in 2012 and 2014 the aboveground biomass (AGB), aboveground necromass (AGN) and belowground biomass (BGB) carbon (C) stocks were measured and C sequestration rates calculated as proxy of ecosystem recovery. The soil organic carbon (SOC) stock to 36-cm depth was also quantified during the 2012 survey. To explore potential drivers of spatiotemporal variation of the forest and páramo C stocks they were related to abiotic and biotic variables. Andean forest C stocks were influenced mainly by disturbance history and tree-species composition. Páramo C stocks´ spatial variation were related to the elevation gradient; we found a positive significant trend in páramo AGB-C stocks with elevation, whereas we found a significant negative trend in AGN-C stocks. Likewise, significant temporal changes were found for AGB-C and AGN-C stocks. Net increases in AGB-C stocks were the largest in the Andean forest and páramo, 2.5 Mg C ha^-1^ year^-1^ and 1.5 Mg C ha^-1^ year^-1^ respectively. Carbon sequestration rates were partly explained by environmental variables. In the Andean forest, plots with low dominance of *Baccharis padifolia* were observed to present higher AGB-C and lower BGB-C sequestration rates. In the páramo, higher sequestration rates for AGB-C were found at higher elevations and associated with higher levels of growth-forms diversity. Temporal changes in BGB-C stocks on the contrary were non-significant. Our results indicated that terrestrial aboveground C sequestration rates might be an appropriate indicator for assessing Andean forest and páramo recovery after human disturbance.

## Introduction

High-elevation tropical Andean ecosystems, such as woodlands dominated by the genus *Polylepis* (hereafter Andean forest) and grass dominated páramo, are of paramount importance due to the economic and ecosystem services they provide [1–3]. These services include diverse and unique biodiversity [4, 5], carbon (C) storage [6–11], and water provision for direct human consumption, irrigation and hydropower generation [8]. However, large areas of the Andean forest and páramo in the Ecuadorian Andes have been degraded by a diverse array of human land-uses including: overgrazing, fire, timber extraction, introduction of exotic species and agricultural activities [8, 12–15].

In the high Andes, ecosystem services, including terrestrial C storage, and threats to those services, are intrinsically interlinked [15–17]. Aerial and belowground C stocks vary greatly along the Andean forest and páramo gradient with the ratio between aboveground biomass (AGB) and soil organic carbon (SOC) decreasing at higher elevations. Typical AGB-C stocks for forest over 3,500 m.a.s.l. (meters above sea level) range between 38–83 Mg C ha^-1^ [18, 19], whereas for páramo these values can be around 4 and 17 Mg C ha^-1^ [20, 21]. Aboveground necromass (AGN) C stocks have been measured considering land use and vegetation cover mainly in forests [9, 22, 23], but the effect of other abiotic (e.g. elevation) variables have not been assessed in páramo ecosystems. Belowground biomass (BGB) C stocks, have been rarely reported for páramo and no clear trend with elevation has previously been documented [7]. High relative humidity, low temperatures and volcanic ash deposition reduce decomposition rates in páramo and Andean forest soils resulting in increases in the SOC stock in the organic layers [24]. Different environmental and anthropic factors may influence C allocation in the different pools of high elevational gradients. For example, in Andean forests selective logging coupled with annual burning events results in ecosystem degradation including reduction in C stock, forest fragmentation and species loss [19]. While in páramo, areas of extensive cattle grazing and associated burning events have homogenized the vegetation (i.e. reduced growth-forms diversity) and resulted in a decrease in AGB and BGB-C stocks [12, 25–27]. Furthermore, in páramo ecosystems consecutive fires and persistent grazing have shown to decrease AGB production [28, 29]. Yet, grazing and burning may have effects on the SOC stock of both páramo and Andean forest ecosystems [1, 8, 14].

Recognition of the societal value of high Andean ecosystems and their ecosystem services, together with the current threat they face due to ongoing land-use change, have led to an increased global awareness of the need to restore the functionality of regional threatened ecosystems to secure human wellbeing [30]. Yet, our understanding on how tropical mountain ecosystems recover from land-use disturbance is still limited, particularly in areas experiencing natural recovery over long periods.

Secondary tropical forest, previously converted into pasture land-use, have been shown to recover vegetation structure and biomass to pre-disturbance levels over an 80-year period [31]. AGB accumulation in recovering secondary forests has been reported to be 5, 3 and 1 Mg C ha^-1^ year^-1^ over the first 5–7, 20 and 80 years of succession respectively [32–35]. Yet, these studies focused on mountain forest of low and mid altitudes whereas high Andean montane forest studies over 3,700 m.a.s.l., particularly in *Polylepis* stands, are very rare. The few available studies (e.g. [19, 36]) are based on chrono-sequence designs (synchronic approach) instead of diachronic approaches in which non-linear secondary succession dynamics can be assessed. To the best of our knowledge there are no published studies assessing ecosystem recovery of *Polylepis* forests using repeated surveys of the same sites.

Natural recovery of páramo vegetation is a slow process, involving diverse ecosystem responses [37, 38]. For example, in *Calamagrostis* spp. dominated páramos AGB-C stocks recovered to its original state five years after a burning event [12]. However, this process could take longer depending on the frequency and intensity of fire events, grazing, original degree of disturbance of the ecosystem, and on the local climatic (i.e. precipitation, humidity, wind velocity and temperature) conditions [12, 39, 40]. Furthermore, natural recovery may be slower at higher elevations [12] and for sites dominated by few species, such as páramo tussock grasses of the genus *Calamagrostis* [29, 41].

Two major knowledge gaps limit our understanding of C storage dynamics in recovering high Andean ecosystems at local scales [18, 42]. The first one is the scarcity of research on the natural recovery process for different C pools along environmental (e.g. elevation) gradients using repeated surveys in the same region. Secondly, although different studies have addressed the natural recovery in C stocks in high Andean ecosystems, primarily páramo, after a disturbance [12, 19, 38, 39, 43–45], only a handful of them have included ancillary variables for explaining C-dynamics during secondary succession [7, 27, 43, 46, 47].

In this paper we seek to improve the understanding of the natural recovery of terrestrial C stocks in both high-elevation Andean forest (> 3,700 m.a.s.l.) and páramo. To do this we provide a comprehensive assessment of AGB, AGN, BGB, and SOC stocks in Andean forest and páramo (northwestern Ecuador) 23 years after grazing, firewood extraction and fire regimes ceased. We use biomass-C sequestration rates as a proxy to infer the recovery status of the ecosystem [29, 41, 48, 49], and tested three hypotheses: (i) Páramo aboveground C stocks (AGB and AGN) and elevation have an inverse relationship, whereas belowground C stocks (BGB and SOC) has no response to increases in elevation; (ii) C sequestration rates for AGB and AGN, in both Andean forest and paramo, are higher than those for BGB; and (ii) areas at lower elevation exhibit higher rates of C sequestration.

## Materials and methods

### Study area

**Fig 1:**
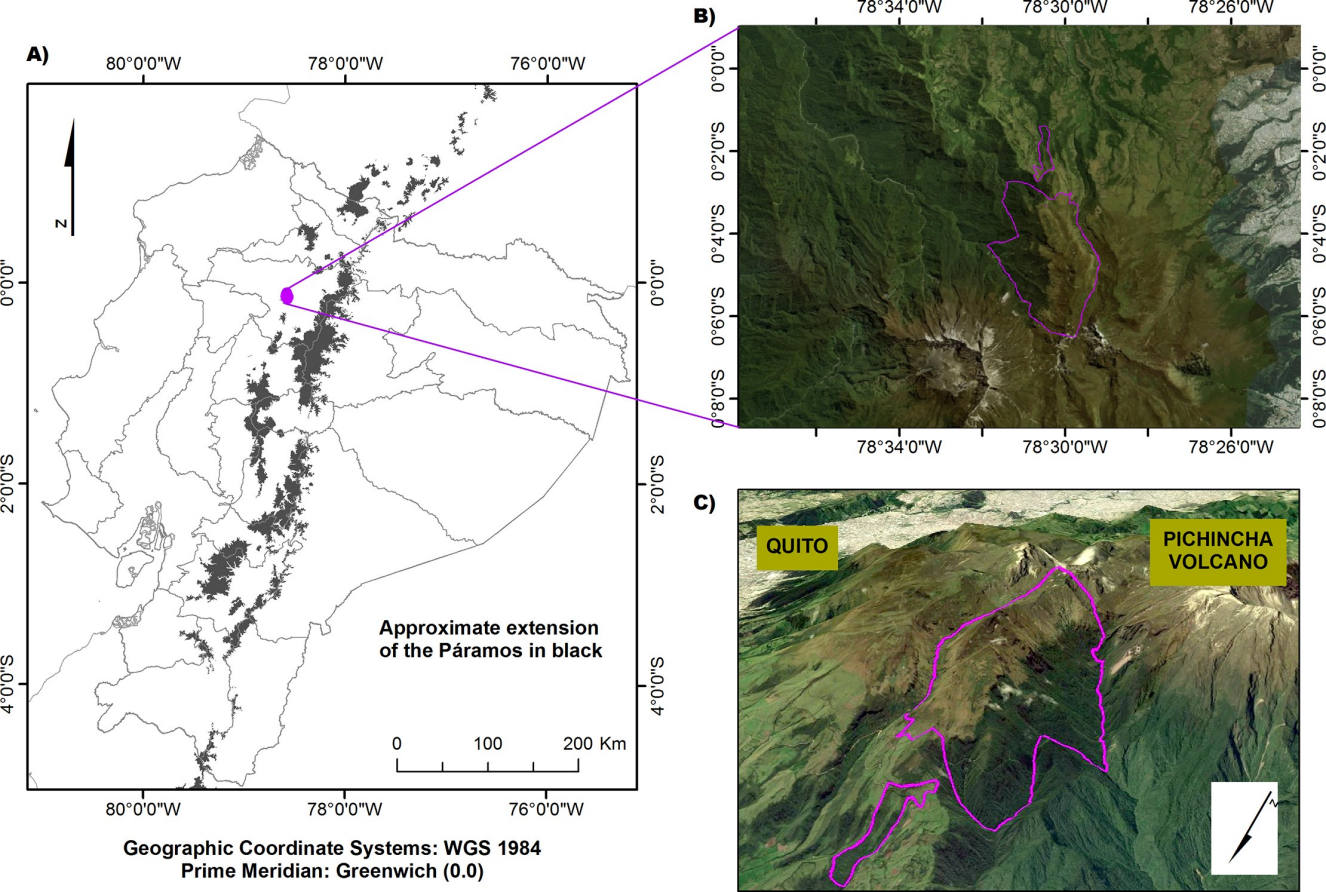
A) Study site in Yanacocha Reserve, Pichincha, Ecuador, B) Aerial view of the Yanacocha Reserve, C) View of the elevational gradient with the two studied ecosystems enclosed by the purple polygons. Source: Google Earth 2012.

The research was conducted in the Yanacocha Reserve (Ministry of Environment Research permit:004–15 IC-FLO-DNB/MA), owned by the Jocotoco foundation. The Yanacocha Reserve has 1,200 ha of extension, which 960 ha are covered by montane evergreen forest and 240 ha by páramo; it is located 5 km northwest of Quito (0.13° S, 78.58° W) and is underlain by the Pichincha volcanic complex. The study area (50 ha, 3,600–4,350 m.a.s.l.; Fig 1) located in a glacial valley, characteristic of the high Andes, has been heavily influence by Quaternary volcanic activity, ~ 1,100–2,000 years ago [50, 51]. The slope angle within the study area is highly variable (from 5° to 26°).

Soils within the Yanacocha Reserve study area are Andosols, typical of the high Andes [52]. Preliminary information obtained from soil pits within the study area (one pit in forest and two in páramo) showed that soils contained well-defined layers of volcanic ash intercalated with the organic layers. The topsoil layer in both forest and páramo was found to vary from 15 to 18 cm depth containing more than 7% of soil organic matter (SOM); it was also composed of litter and an array of fine, medium and coarse roots. The organic layers below the volcanic layers were found to be of similar thickness than those in the topsoil, although the proportion of root biomass was found to decrease with increase in depth. Roots were present up to 155 cm in forest and 55 cm depth in páramo.

The lower elevation limit of the study area within the Yanacocha Research is ca. 3,600 m.a.s.l., about 300 m below the upper forest line. At the upper forest line, the species composition is characterized by non-deciduous trees like *Polylepis pauta*, *Gynoxys acostae*, *Escallonia myrtilloides*, *Baccharis padifolia*, *Columellia oblonga* and *Buddleja incana* [53]. The páramo starting at 3,900 m.a.s.l. is characterized by grass species like *Calamagrostis intermedia*, shrubs like *Pernettya prostrata*, and herbs like *Hieracium frigidum* or *Lachemilla orbiculata*. At higher elevations the dominant growth-forms shift from grasses to sclerophyllous shrubs (including *Chuquiraga jussieui*, *Loricaria thuyoides* and *Baccharis caespitosa*) and, prostrated herbs and cushions mats (including *Werneria nubigena*, *Xenophyllum humile*, and *Plantago rigida*) [54]. Both forest and páramo ecosystems in the Yanacocha Reserve have a history of human disturbance [55]. Extensive cattle grazing combined with annual burning events transformed the area and almost obliterated the shrubby elements of the páramo, and reduced the upper forest line to at least 100 meters in the most exposed areas of the reserve. Selective logging and firewood extraction degraded the Yacocha Andean forest (personal communication with local inhabitants). However, most human activities (grazing, fire and logging) ceased in 1995 when the reserve was created, leaving the ecosystems to naturally recover (personal communication with local inhabitants). Unfortunately, there are no records of C stocks and species or growth-forms composition along the elevation gradient at the time of the cessation of human activities.

**Fig 2:**
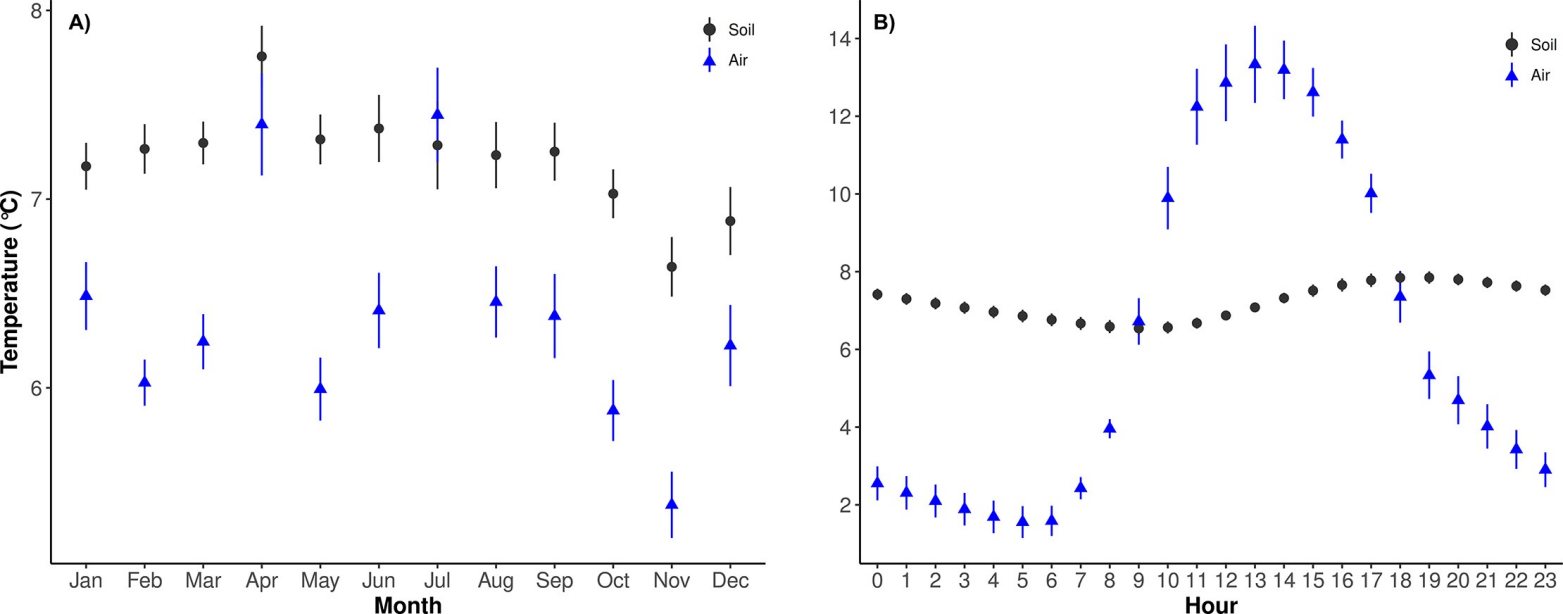
Mean monthly (A) and hourly (B) air (n = 9, blue arrows) and soil (n = 13, black dots) average temperature and mean standard errors for the period November 2013–October 2014. Data from I-button sensors installed in random plots along the gradient.

The climate of the Yanacocha Reserve is typical of high tropical mountains areas [56]. Average hourly temperature values over a 24-hour cycle from November 2013 to October 2014 show a more pronounced variation than the monthly average variation, e.g. air temperature at 10 cm aboveground can vary by 2–10°C in one day (Fig 2B) compared with monthly variations, of 6.5–8°C (Fig 2A). Soil temperature, at 10 cm in depth, is comparably stable through both daily and yearly cycles (6.5–8°C daily, 6.7–7.7°C yearly).

### Experimental design

Two censuses were conducted to quantify the spatiotemporal increases of terrestrial biomass C stocks, as a proxy for ecosystem recovery. The baseline was determined during the months of June to December of 2012, and the second census was conducted during the same months in 2014. Within the 50-ha study area of the Yanacocha Reserve thirty-nine square permanent plots (sample units) of 10 x10 m size were established. Ten plots were located in the forest and twenty-nine in the páramo. The plots were deployed in a stratified random design, where each ecosystem was considered as a stratum. We used a preliminary C stocks’ census in páramo and a forest inventory for defining the number of plots to be deployed in the field. The number of plots for each ecosystem was calculated as follows: n = (ns^-1^) x ((t) (SD) x (E)^-1^)^2^ [57], where *n* is the sample size; *ns* the number of sub-sampling units, four in this case; *t* the sample statistic from the t-distribution for the 95-percent confidence level; *SD* the standard deviation of the sample; and, *E* the allowable error or the desired half width of the confidence interval. Páramo plots were differentiated by elevation, i.e. plots located between 3,900–4,100 m.a.s.l. and plots located between 4,100–4,300 m.a.s.l. The elevation range over which the plots were deployed was designed to capture the gradual transition from the páramo to the superpáramo ecosystem [58, 59].

**Fig 3:**
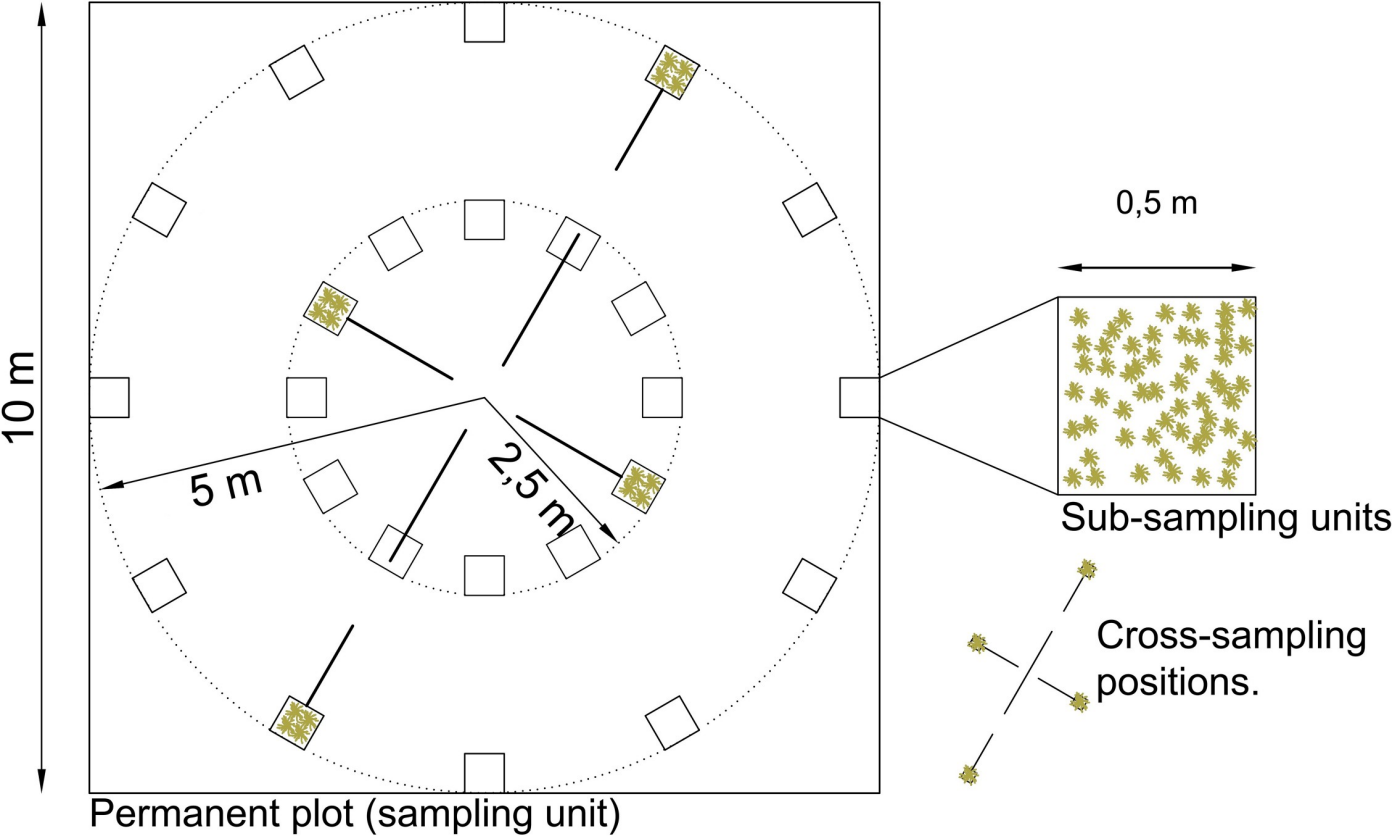
Sampling design in each permanent plot (sampling unit). Four sub-sampling units (filled boxes) are sampled in each census following the cross-sampling positions.

The permanent plots were established facing north, and the corners and center were marked with PVC tubes. Inside each plot the following variables were measured: elevation, coordinates of the center of the plot, slope angle, aspect, AGB, AGN (ground litter and detritus), BGB and SOC in a depth increment of 18 and 36 cm. Each permanent plot was sampled using the Calderón, Romero-Saltos [55] protocol designed to sample terrestrial C stocks destructively through time but with minimum long-term impact. The design provides 12 possible sub-sampling positions in two concentric circles (Fig 3). For each census four sub-sampling units (each 50 x 50 cm) were randomly selected in a cross formation, two from the inner and two from the outer circle. The same procedure was performed for the re-census but with the previously sampled positions excluded from possible selection, i.e. the same positions could not be sampled twice allowing the C stocks to recover in the measured pools.

### Field methods

#### Aboveground biomass sampling

A nondestructive inventory was conducted inside each permanent forest plot for all stems with diameter at breast height (DBH) ≥ 5 cm. To develop site-specific allometric biomass equations, 36 trees from different diameter classes, DBH 5.5–23.3 cm, were collected outside the permanent plots. Trees of four species were harvested based on their prevalence inside the permanent plots: *Polylepis pauta* (21 individuals), *Baccharis padifolia* (4 individuals), *Escallonia myrtilloides* (5 individuals) and *Gynoxis acostae* (6 individuals). Measurements of trunk diameter and total height were recorded for each tree before harvest. Trees were harvested from the base of the trunk. Trunk material, branches and foliage were cut in small fragments for laboratory processing. Wood density was estimated by taking wood cores from the trunk using an increment borer [60].

In páramo, a treeless ecosystem, the AGB of the grasses and shrubs was collected inside each of the four sub-sampling unit within each permanent plot; the crowns and existent stems were harvested, weighted and packed for laboratory processing.

#### Aboveground necromass, belowground biomass and soil sampling

In páramo and forest, AGN, BGB and SOC, were sampled in the same four sub-sampling units within each plot. First, AGN was collected manually leaving the sub-sampling area free of dead vegetation (Ho and H1 horizons were removed), then two soils samples were extracted with a borer, in two depths 0–18 and 18–36 cm from the A horizon. To define the depths of measurement three soil pits were dug along the gradient where soil horizons were visually identified and their depth measured. Eight soil samples, four at each depth, per permanent plot were taken for the analysis of BGB fine roots, ≤ 2 mm, and SOC. We set the 2 mm threshold for separating fine and coarse roots following accepted definitions used by other ecological studies in high Andean and tropical ecosystems [9, 22, 61, 62]. Despite that coarse roots were not considered, the inclusion of a threshold allowed us to compare our BGB results between ecosystems.

SOC was only analyzed in the first census and was assumed to not change significantly over the two years of the study [63].

#### Vegetation and soil coverage

Growth-form and substrate cover was assessed inside each sub-sampling unit in the plots located in páramo to characterize responses of terrestrial C stocks to functional diversity and soil substrate, following the GLORIA protocol [64]. Following an adapted version of the growth-form classification defined by Ramsay and Oxley [56], the percentage cover of each growth-form and substrate was also visually estimated in each plot. Growth-forms were defined as rosette (including stem, basal and acaulescent rosette), tussock, cushion, shrub (upright and prostrate shrub), herb (erect and prostrate), erect grass and cespitous grass. Substrate types were defined as rock, scree, bare soil, aboveground decomposing necromass and bryophytes-lichens.

### Laboratory analyses

Fresh samples (live and dead vegetation, roots and soil) were weighted in the field following collection before they were taken to the laboratory for dry weight quantification. AGB, AGN and BGB samples were dried at 60°C in an oven until stable weight was reached.

SOM was estimated by loss-on-ignition method; the soil samples (roots and rocks were manually separated) were heated at 350°C for 24 h and weighted to quantify the percentage of SOM. We used Kopecky’s rings to collect undisturbed soil cores for determining their bulk density. The soil samples were dried in an oven at 105°C for about 18–24 hours [65]. Prior to these analysis (except bulk density), roots were manually extracted from soil cores during 60 min time following the Metcalfe [66] protocol. Sampled roots were rinsed in water to remove soil particles, oven dried at 60°C in the lab until constant mass was reached, and then weighted.

The immersion method was used to determine the wood density of the core samples [60], these were plunged in water for two hours, then oven-dried to 60°C until a stable weight was obtained (around two days).

### Data analyses

#### Allometric biomass equations

For forest plots, we developed mixed-species allometric models to estimate AGB using trunk diameter, total height and wood density as predictors (see S1 Appendix). Different linear and nonlinear regressions models were tested. Best-fit model was selected by comparing the Bayesian information criterion (BIC), the Akaike information criterion (AIC), the coefficient of determination (R^2^) and the Root mean squared error (RMSE). The first two criteria penalize the number of parameters used in the regression [67], the R^2^ coefficient is the proportion of the AGB variance explained by the model, and the RMSE shows the sum of all regression errors. These four criteria provide enough information to evaluate the performance of the regression model [68]. Based on these analyses, a nonlinear regression model was developed to estimate the AGB in the forest of the Yanacocha Reserve using only the trunk diameter (cm) (to reduce intrinsic errors in the field estimation of tree height) (Equation 1) AGB = 0.041 (DBH) ^2.56^, R^2^ = 0.919, as predictor variable (further details are presented in S1 Appendix).

#### Calculation of carbon stocks

To estimate the mean AGB, BGB, AGN and SOC C-stocks for each permanent plot, the values of all the samples per C pool were averaged. The C stocks were then estimated as 50% of the dry weight of AGB, AGN and BGB pools [69, 70]. Forest AGB for each permanent plot was estimated using the trunk diameter allometric biomass equation derived for the forest (Equation 1). The “Van Bemmelen” factor (= 1.724) was used to convert SOM into SOC [71, 72] as no local information on SOC was available. The prevalence of growth-forms and substrate cover in the four sub-sampling units of the páramo plots were averaged to obtain a mean value for each permanent plot. Growth-form cover was used to calculate a diversity index, Shannon-index, for each plot [73].

Carbon stocks were extrapolated to Mg C ha^-1^ and averaged to estimate their mean values (and their standard deviation) by ecosystem and along the elevation gradient.

#### Carbon sequestration rates

Since mean C stocks of both censuses came from the same permanent sampling units (non-independent), a paired t-tests with unequal variances were performed between censuses to assess significant differences of C stocks on each pool. Normality of the data was first checked using a Shapiro-Wilk test [74].

An ordination analysis was performed to characterize C variation responses to biophysical variables (besides elevation), such as slope, growth-form diversity measured with a Shannon-index, soil substrate (only organic matter, and bryophytes and lichens were used in the analysis as most of the substrates were zero) and species abundances (only for forest). A distance-based redundancy analysis (db-RDA) was performed, since the explanatory and response variables were expected to have a linear relationship, on the environmental, log-transformed, and response variables [75]. This analysis was done separately for the páramo and for the Andean forest, because the set of environmental variables expected to control C sequestration rates was different for each case. For the Andean forest, the basal area per species was calculated to determine how much variation in the C stocks at the plot scale could be explained by tree community composition. Manhattan distance was used to interpret the influence of environmental variables on C stocks variations [75], and the adjusted R^2^ (R^2^-adj) for assessing the proportion of the C stocks variance explained by the independent variables and penalized by the number of parameters included the model that do not improve its performance. All the statistical analyses were conducted in R [76], code included in the ‘vegan’ package was used to perform the ordination analyses [77].

## Results

### Carbon stocks of the different pools from Andean forest and páramo

**Fig 4:**
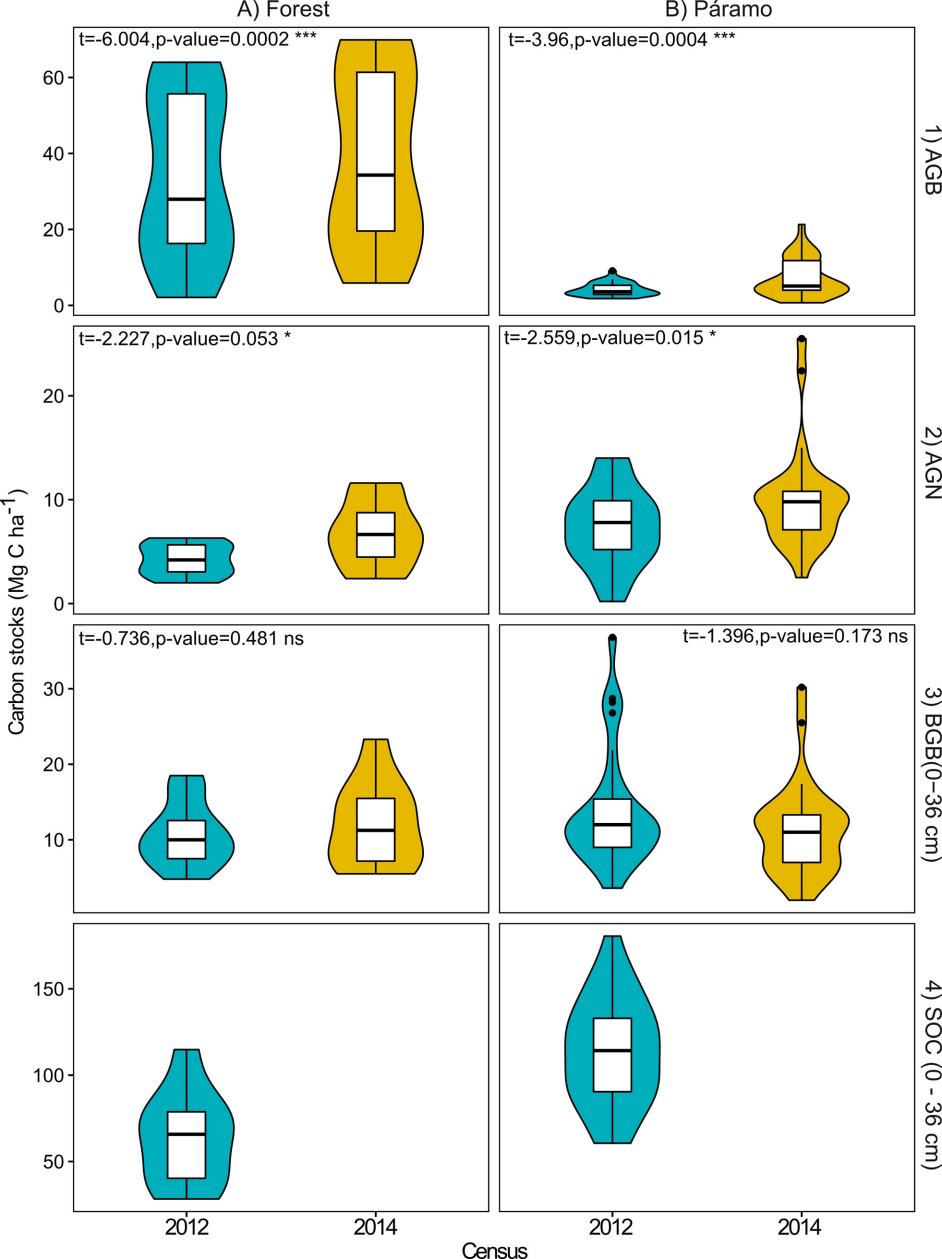
Violin plots of the carbon stocks (Mg C ha^-1^) in the sampled pools in the forest (A) and páramo (B) ecosystems for 2012 and 2014, Yanacocha Reserve, Pichincha, Ecuador. 1) AGB: aboveground biomass, 2) AGN: aboveground necromass, 3) BGB: belowground biomass, and 4) SOC: soil organic carbon. SOC samples were not taken in 2014 because of the slow soil dynamics. BGB-C and SOC stocks were taken at 0–36 cm depth.

**Fig 5:**
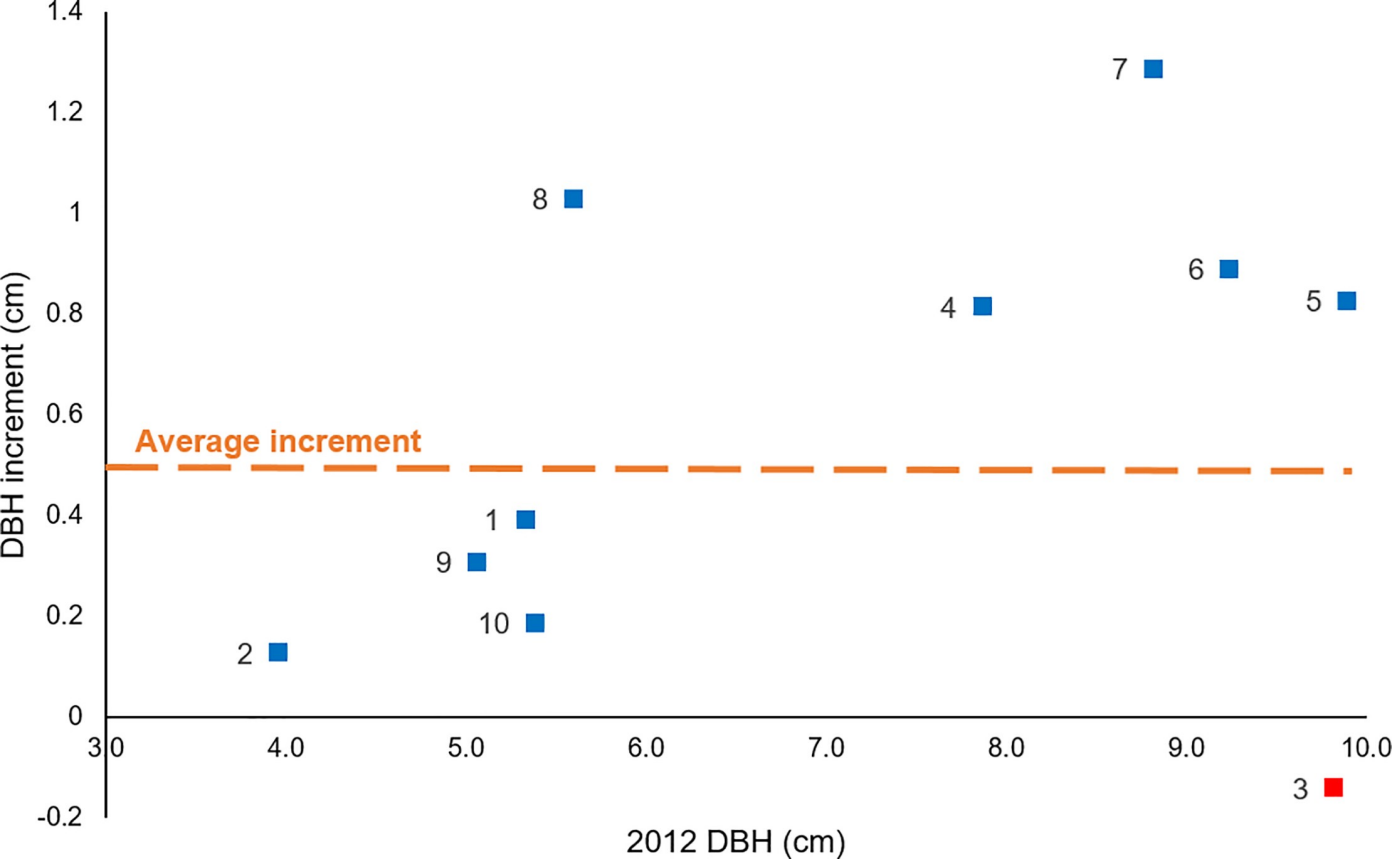
Diameter at breast height’s (DBH) increment (cm) per plot in the forest against 2012 DBH (cm). Blue squares represent the forest plots with positive DBH increment, red squares represent those with decreasing DBH. Orange dashed line is the average DBH increment among all forest plots, 0.5 cm.

There were significant differences between the 2012 and 2014 for AGB and AGN-C stocks (Fig 4A1, 4B1, 4A2 and 4B2). AGB-C content in the Andean forest was 34 (±22) and 39 (±24) Mg C ha^-1^ in 2012 and 2014 respectively. We found an increase in the number of individuals recruited, and the total number of individuals, in each DBH class of trees in 2014, except for the 2.5–4.9 and the 5–10.9 cm classes that experienced a subtle decrease in number of individuals (Table 1). Furthermore, a small increase in DBH among all trees was observed between the two censuses, 0.5 cm (t = 1.853, p = 0.033). Similarly, we recorded increases in all the tree DBH classes, and by 0.1–1.3 cm among plots. DBH did not increase in one plot (t = -0.0922, p = 0.537) (Fig 5). The relatively high variation in plot-based AGB was influenced principally by the DBH profile of trees in each plot (see S1 Fig). Only half of the Andean forest plots had trees with DBH greater than 20 cm, mostly *Polylepis pauta*, which contributed more than 35% to the AGB. On the other hand, young trees with DBH of 5–10 cm, which represented 27–96% of the individuals in a plot, contributed nearly 17% to the total forest AGB in both years. Furthermore, the DBH profile of the plots without large adult trees (DBH > 20 cm) was highly heterogeneous and dominated by juvenile trees (DBH < 5 cm) (see S1 Table).

**Table 1:** Individuals distribution by diameter classes in 2012 and 2014 of the Yanacocha Reserve Andean forest plots (n = 10).

Diameter class (cm)	# Individuals		Average trunk diameter (cm) (±1 SD)	
	2012	2014	2012	2014
**2.5–4.9**	329	276	3.5 (±0.7)	3.6 (±0.6)
**5–10.9**	214	209	6.9 (±1.5)	7 (±1.5)
**11–20.9**	72	80	14.4 (±2.6)	14.5 (±2.7)
**>21**	15	16	25.1 (±3.4)	25.3 (±3.6)
**Total**	630	581		

**Fig 6:**
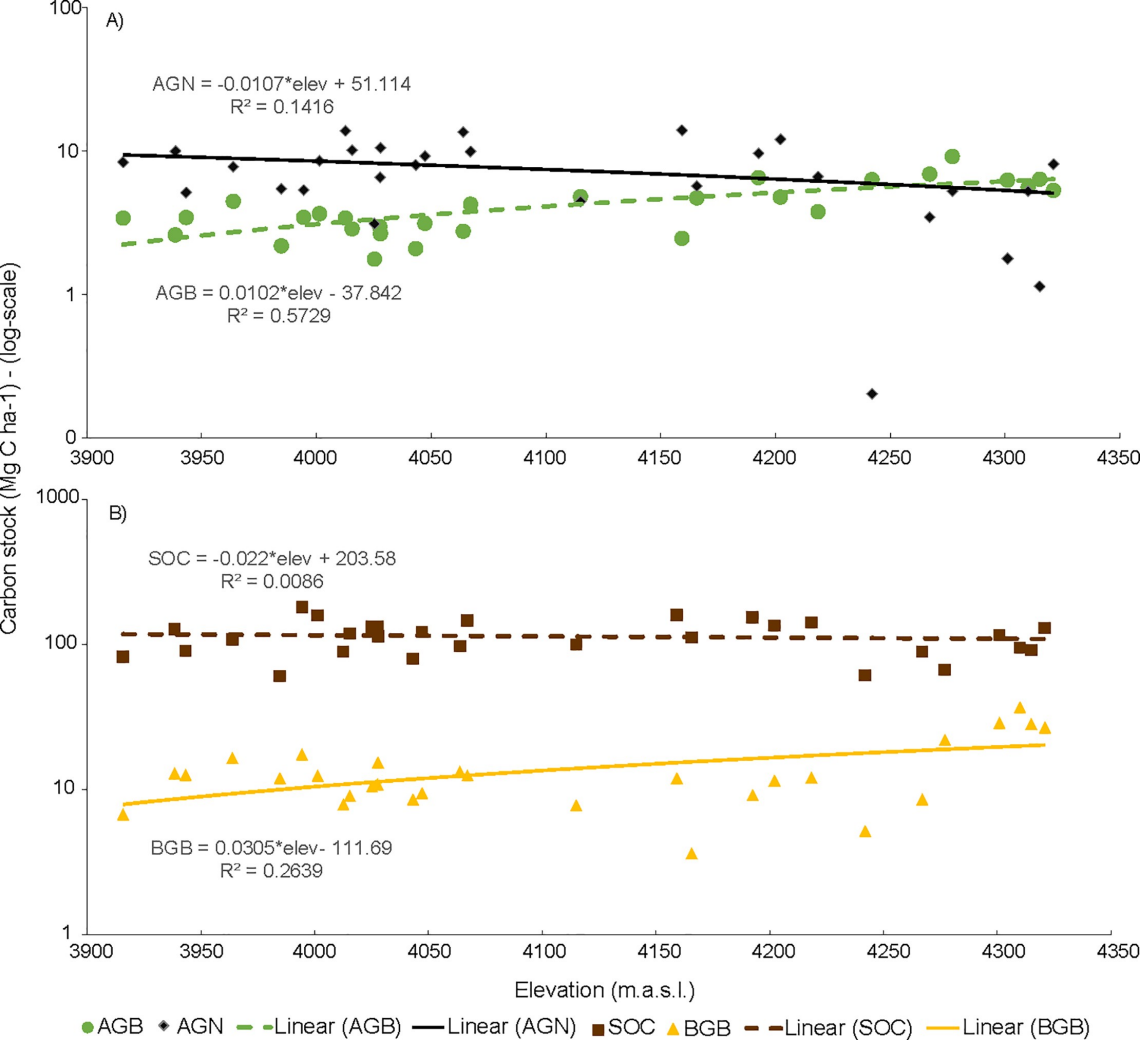
2012 C stocks variation in the elevational páramo gradient. A) Aboveground C pools (AGB, green circles, and AGN, black diamonds); b) Belowground C pools (BGB, yellow triangles, and SOC, brown squares). For visualization purposes y-axis is presented in a log-10 scale.

AGB was the smallest C pool in páramo, 4 (2012) and 7 (2014) Mg C ha^-1^, whereas the largest pool was SOC, 113 Mg C ha^-1^. Páramo AGB-C stocks increased with elevation (p = 2.02x10^-6^, R^2^ = 0.57). AGN was the smallest C pool in the forest, ca. 4 and 7 Mg C ha^-1^ for the 2012 and 2014 sampling, respectively. In the páramo, the AGN-C pool decreased slightly as elevation increased (p = 0.04, R^2^ = 0.14) (Fig 6A).

Non-significant differences between samplings were found in BGB-C stocks in the Andean forest and páramo. BGB-C stocks had a mean value of 11 Mg C ha^-1^ in 2012 and 12 Mg C ha^-1^ in 2014 in forest (t = 0.7358, p = 0.4806), whereas in páramo these values were higher in 2012, 14 Mg C ha^-1^, and lower, 11 Mg C ha^-1^, in 2014 (t = 1.398, p = 0.1731) (Fig 4A3 and 4B3). Interestingly, we found a significant increase of páramo’s BGB-C stocks along the elevational gradient. However, 74% of the variance in BGB-C stocks was not explained by the elevation (p = 0.004, R^2^ = 0.26) (Fig 6B). SOC stock in the Andean forest at 0–18 cm depth was higher than that at 18–36 cm depth, i.e. 38 *vs*. 26 Mg C ha^-1^ in 2012. SOC in páramo did no vary along the elevational gradient (p = 0.63, R^2^ = 0.009) (Fig 6B).

AGN had the higher relative increase in both ecosystems, 84% in the forest and 202% in the páramo. Similarly, the AGB-C stock presented the highest absolute increase in both ecosystems: 5 and 3 Mg C ha^-1^ in the Andean forest and páramo, respectively. On average, the absolute relative variation in C stocks, not including SOC, in páramo was higher than in the Andean forest, 89 and 48% respectively.

### Effects of biotic and abiotic variables on carbon sequestration rates

**Fig 7:**
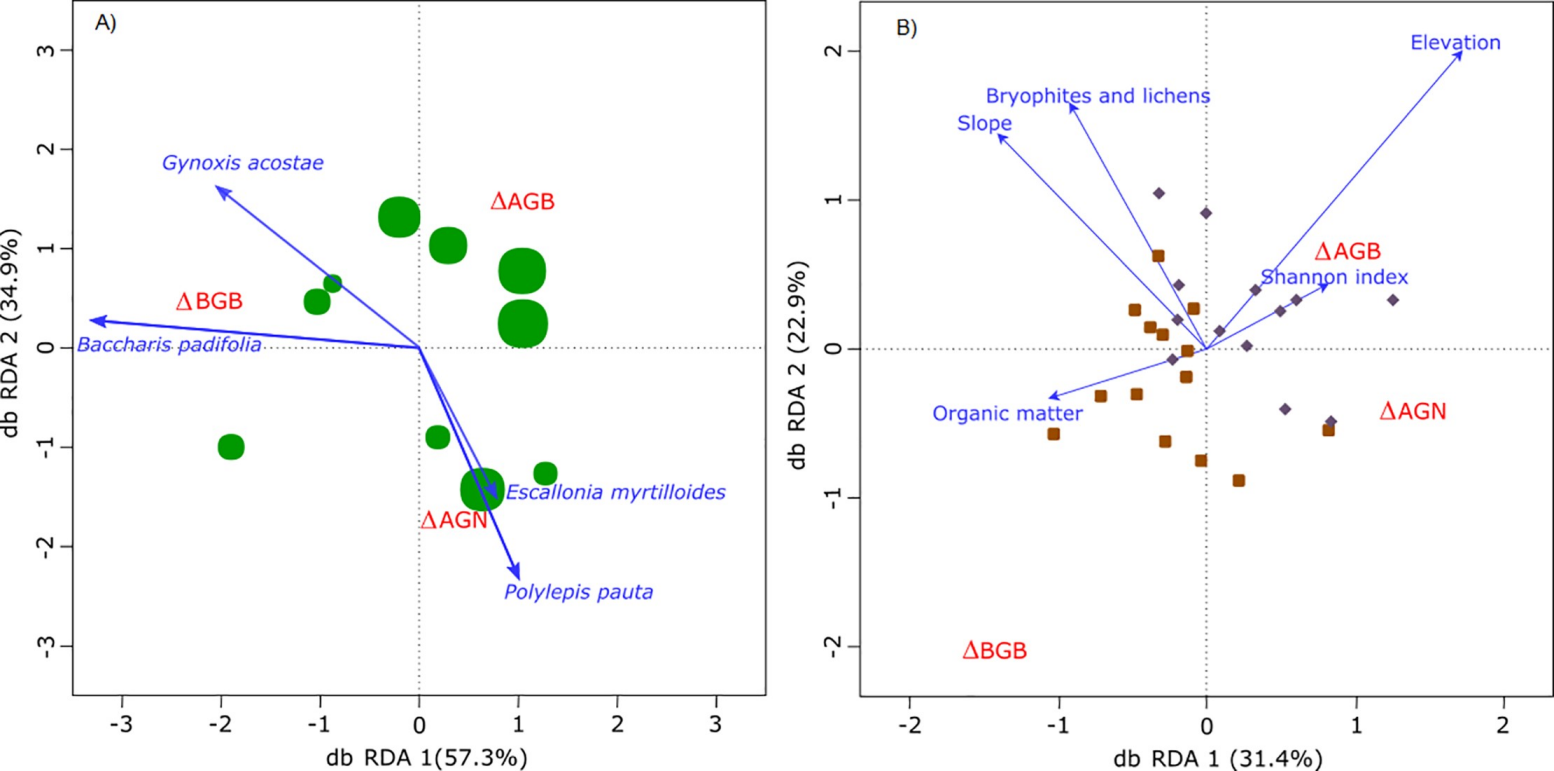
Biplots of distance-based redundancy analyses of the environmental and carbon stock differences for A) Andean forest and B) páramo. Response variables (carbon stock differences) and sites are presented in red and blue text respectively. Andean forest plots, green circles, below 3,900 m.a.s.l.; the size of the circle is relative to the plot’s mean trunk diameter in the first census. Páramo plots represented with a diamond are located above 4,100 m.a.s.l. and with a square between 3,900–4,100 m.a.s.l.

The db-RDA conducted to assess the effect of the biotic and abiotic variables over the differences in biomass and necromass-C stocks showed two distinctive behaviors in the forest model and in the páramo model. For the forest model the R^2^-adj coefficient was 0.29, i.e. more than 70% of the C stocks variance was not explained by the included variables. Only *Baccharis padifolia* was significant to the model, influencing BGB gains (Table 2). Plots with higher DBH are ordinated in the top right quadrant of the biplot presenting higher AGB gains (Fig 7A). AGN gains could not be explained with the employed environmental variables because of their non-significance in the model. We did not include elevation as an ancillary variable as the gradient in the forest is too short to identify an effect of this variable over the C differences in all the analyzed pools.

**Table 2:** Loads, eigenvalues and significance level of the distance-based RDA including the environmental variables analyzed for testing the influence over carbon stock differences for both ecosystems, Andean forest and páramo.

Model	Environmental variables	Loads		Permutation test		
**Andean forest**		**db-RDA1**	**db-RDA2**	**Variance**	**F**	**Pr(>F)**
	***Baccharis padifolia***	**-0.97**	-0133	3.9347	3.305	0.04
	***Escallonia myrtilloides***	-0.183	-0.42	0.763	0.641	0.61
	***Gynoxys acostae***	-0.525	-0.461	0.256	0.215	0.917
	***Polylepis pauta***	-0.263	**0.713**	2.244	1.885	0.154
	**Eigenvalue**	4.12	2.508			
	**Proportion explained**	57.3	34.9			
	**R**^**2**^	0.61				
	**R**^**2**^**-adjusted**	0.29				
**Páramo**	**Elevation**	**0.5398**	**0.7247**	36.522	5.724	0.004
	**Slope**	**-0.5678**	0.4867	9.464	1.483	0.244
	**Organic matter**	-0.3399	-0.1167	30.359	4.758	0.014
	**Shannon-index**	0.2308	0.1599	27.516	4.312	0.012
	**Bryophytes and lychens**	-0.4024	**0.5325**	12.15	1.904	0.156
	**Eigenvalue**	0.874	0.637			
	**Proportion explained**	31.42	22.87			
	**R**^**2**^	0.44				
	**R**^**2**^**-adjusted**	0.32				

Bold values represent maximum load values, grey boxes represent the statistically significant variables to each model.

In the páramo model the R^2^-adj was 0.32; nonetheless, the Elevation, Organic matter and Shannon-index were significant to the model (Table 2). These three variables ordered half of the plots in two quadrants, upper right quadrant and left lower quadrant (Fig 7B). Elevation and Shannon-index are directly correlated with higher AGB gains (plots located above 4,200 m.a.s.l.). On the other side, less diverse plots located at lower elevations with high organic matter contents (plots located between 3,938–4,160 m.a.s.l., see S2 Appendix) presented lower gains in AGB but higher gains in BGB. The other two variables: slope and bryophytes-and-lichens’ percentage coverage had a small influence on AGN gains.

## Discussion

We have quantified biomass and necromass-C stocks in 2012 and 2014 in the Andean forest and páramo in the Yanacocha Reserve. In the 2012-census the total average C stock estimated for the Yanacocha Reserve was 132 Mg C ha^-1^ with the SOC (0–36 cm depth) pool being the largest terrestrial C stocks in the páramo, 113.4 Mg C ha^-1^, and Andean forest, 63.4 Mg C ha^-1^, respectively. Increases between 2012 and 2014 in AGB-C stocks were observed to be the largest, with a mean 1.5 Mg C ha^-1^ increase both in the forest and páramo.

These overall data demonstrate that the Yanacocha Reserve has acted as a C sink in the AGB C pools over the studied period. However, spatial and temporal variation between ecosystems and carbon pools was also observed. In Andean forest C stocks were influenced mainly by disturbance history and on the tree-species composition, whereas in páramo spatial variation C stocks were related to the elevation gradient in most of the pools, partially confirming our first hypothesis. Temporal variation in AGB and AGN between the two years (2012–2014) suggests that the C sequestration rates were higher in the aboveground pools and were associated with higher elevations and plant functional diversity, confirming our second hypothesis and contrary to our third hypothesis.

### Carbon stocks’ spatial variation

The spatial patterns of C stocks along the páramo elevation gradient, partially confirmed our first hypothesis. However, contrary to expectations, we found a positive significant increase in páramo AGB-C stocks with elevation, whereas we found a significant opposite trend in AGN-C stocks (Fig 6A). The shift along the elevation gradient in vegetation composition explained the majority of the spatial variance in AGB-C and AGN-C stocks, whereas variations in BGB-C stocks were, only, partially dependent on elevation. We found no changes in SOC-C stocks with elevation. This lack of change is likely to be because the elevational range studied (3,900–4,250 m.a.s.l.) was not sufficient to capture a major change in soil climate which is thought to be the major driver of variation in SOC-C stocks [78]. Yet, the significant changes of most C-pools along the elevation gradient in the Páramo, suggests the relevance of elevation as a primary factor controlling spatial patterns of carbon allocation across time.

**Fig 8:**
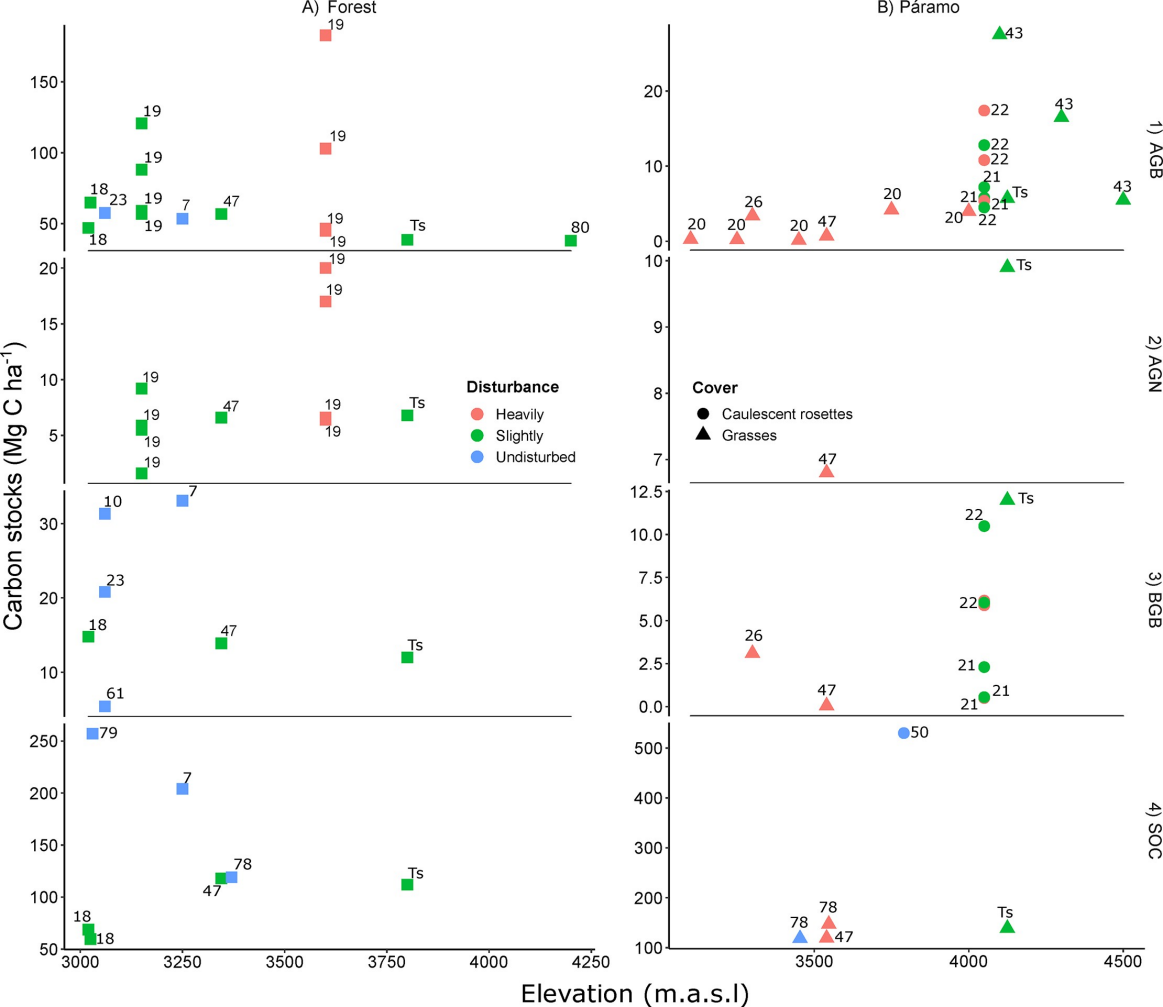
C stocks comparison among published studies in high Andean ecosystems for forest (a) and páramo (b) ecosystems. Reported values by C pool, AGB (1), AGN (2), BGB (3) and AGN (4) have been categorized according to the reported site degradation status: undisturbed (blue fill), slightly (green fill) and heavily disturbed (red fill) sites. In addition, for the páramo sites we have included their reported growth-form cover: caulescent rosettes (circles) and grasses (triangles). Sites in forest ecosystems are represented as squares. The number besides each observation is the reference to the study where ‘Ts’ refers to this study.

On average, in 2012 the páramo stored more C (138.7 ± 32.8 Mg C ha^-1^) than the forest (112 ± 26.8 Mg C ha^-1^) mainly due to differences in the size of the SOC pool (0–36 cm depth) (Fig 4A4 and 4B4). The finding that páramo ecosystems store more carbon than Andean forest was in line with previous studies [47, 78]. The total average C value reported here (125.4 Mg C ha^-1^) is somewhat lower than the value reported by Gibbon, Silman [47] across a similar, non-disturbed, transition in the Eastern Peruvian Andes (161 Mg C ha^-1^) and may reflect the different disturbance histories (Fig 8A1).

Carbon stock allocation was different between the forest and the páramo at Yanacocha. While nearly 57% of the C stock in the Andean forest was allocated to the SOC pool, this allocation was higher in the páramo, 82%, in line with other studies [9, 79]. Previous studies (this included) indicate that the SOC pools in the páramo and in the Andean forests store at least 50% of the total C stock [18, 19, 47], and potentially as much as ca. 90% [80].

### AGB-C stocks

AGB-C stocks values found in both forest (33.7 Mg C ha^-1^) and páramo (4 Mg C ha^-1^) ecosystems in the Yanacocha Reserve are at the lower end of values reported from other studies [23, 80], probably related to the early successional stage of the Andean forest after land-use change in combination with the high elevation where it is located (Fig 8A1 and 8B1). For instance, the majority of measured trees across the 10 plots had a DBH < 10 cm and stored only 17% of the total AGB-C stock found in the Yanacocha forest (Table 1). Conversely, studies reporting higher AGB-C stocks [10, 18, 19, 47] than the Yanacocha Reserve were located at lower elevations, had less human impact, and affected by different local environmental factors (Fig 8A1). Taken together with previous studies, our results suggest that low elevation range, gentle slopes and an homogeneous precipitation throughout the year appear to be major contributors to higher forest AGB-C stocks. However, methodological factors, e.g. plot size and employed allometric equations, used for measuring AGB-C might be influencing the variation in the reported AGB-C stocks across studies. The specific effects of these factors can be found elsewhere in the literature [81–84].

Average páramo AGB-C stocks found at Yanacocha (5.7 Mg C ha^-1^) were lower when compared with undisturbed cases (i.e., 17.4 Mg C ha^-1^), and similar to those reported in studies developed in degraded páramo ecosystems (i.e., 4 Mg C ha^-1^; Fig 8B1). Furthermore, we found a significant increase of AGB-C stocks with elevation, as opposed to expected, partially explained by the plot’s vegetation composition (Fig 6 and Fig 8B1). A gradual shift in the dominance of growth-forms, from tussock grasses to prostrate and erect shrubs, at mid-elevation range, to cushion matts and basal-rosettes at high elevations (see S2 Appendix). Stem-rosettes and tussock grasses, both showing positive relationship with higher AGB-C stocks, were also the dominant growth-forms in the Colombian locations reporting high AGB-C stocks (Fig 8B1). The differences in human impacts among higher and lower plots could also explain the increased AGB-C stocks along the elevation gradient; it is possible that plots at higher elevations were less disturbed by human use. When compared to other studies, higher páramo AGB-C stocks were related to undisturbed study cases and located in a lower elevation range (3,450–4,100 m.a.s.l.), whereas lower AGB-C stocks were primary related to land use impacts and differences in the vegetation composition (e.g. dense tussock grasses vs. stem-rosettes (Fig 8B1)).

### AGN-C stocks

AGN-C stocks in the Yanacocha Reserve forest (4.2 Mg C ha^-1^ in 2012) were lower than in the páramo (7.3 Mg C ha^-1^ in 2012), and within the range reported in other studies (Fig 8A2 and 8B2). Apparently, from the few available cases, AGN-C stocks are controlled primary by land use disturbance regimes. A peak in the AGN-C stocks has been reported in recovered forests with mid-range values of the AGB distribution [85]. Beyond that peak, as AGB-C increases, the proportion of AGN-C decreases. Thus, we expect that over the years, as AGB-C stocks increases, the opposite will occur with the AGN-C stocks.

In the Yanacocha páramo the AGN-C stock was higher than values previously reported for similar ecosystems (Fig 8B2). Higher C stock values of AGN found in our study could be related to the dominance of tussock grasses in the majority of the páramo plots (see S2 Appendix). According to Monteiro, Hiltbrunner [86] high necromass and litter values are particularly characteristic of tussock graminoids such as those that are the dominant growth-form in the lower and middle section of our study area. The dominance of tussocks in the páramo at Yanacocha likely reflects the characteristic vegetation successional stage after the suppression of fire and grazing disturbance [12, 16, 21].

#### BGB-C stocks

BGB-C stocks, excluding coarse roots, were the only stocks that did not differ between ecosystems (t = 1.1986, p = 0.238). An average of 12 Mg C ha^-1^ was found in both Andean forest and páramo. The Andean forest BGB-C stocks at Yanacocha (11 and 12 Mg C ha^-1^ in 2012 and 2014 respectively) are in the range of other reported values for equivalent sites, 5–15 Mg C ha^-1^ (Fig 8A3). However, previously published BGB-C stock values for páramo grasslands report considerably lower values (0.5–3.1 Mg C ha^-1^) than that found at Yanacocha (Fig 8B3). The differences in BGB-stocks along the elevational gradient in the páramo are likely due to methodological constraints and their intrinsic uncertainty. For instance Gibbon, Silman [47] used a mean root to shoot ratio for estimating the C stocks in roots, which might underestimate the BGB-C stocks. Other causes may be related with plant composition, e.g. the Eynden [26] study was performed in puna grasslands dominated by *Calamagrostis* spp. containing more AGB-C than BGB-C stocks. Albeit methodological constraints, our results of the BGB-C stocks suggests that the potential for BGB root pools to be a more important one in many high-elevation systems than previously reported. Improved methods such as including coarse roots (˃ 2 mm) and continuous monitoring could help elucidate this critical knowledge gap.

#### SOC stocks

The SOC stocks (0–36 cm depth) at the Yanacocha Reserve, 112 and 139 Mg C ha^-1^ for the Andean forest and páramo respectively, were similar to previous studies (Fig 8A4 and 8B4) [10, 50, 61, 87]. The difference between forest and páramo at Yanacocha is likely exacerbated by the historical fire events that are known to have occurred in the forest. Román-Cuesta, Salinas [45] reported differences in SOC in burned and unburned forests in the Southern Peruvian Andes. The low SOC stocks associated with past fire events indicates that the detrimental impact of burning on terrestrial ecosystem C storage may have a long below-ground legacy. Aside from the importance of fire, several other environmental factors are known to affect SOC (i.e. land cover, temperature, moisture and radiation) but those specific factors where not assessed in this study [8, 78, 88]. Further studies are needed to understand the effects that microhabitat conditions have over SOC stocks.

#### Carbon stocks temporal change due to natural recovery

We recorded significant differences in C stocks between both years in both ecosystems, with AGB and AGN-C stocks in 2014 being higher than those in 2012. Similarly, our results suggest that C sequestration rates are higher in the AGB and AGN, in comparison with BGB along the Yanacocha Reserve elevational gradient, confirming our second hypothesis.

#### Aboveground-C stocks’ recovery

AGB-C increases in the Yanacocha Reserve were observed both in the forest and in the páramo ecosystems (Fig 4A1 and 4A2). The AGB-C increase rate was 2.5 Mg C ha^-1^ year^-1^ in the forest (see S3 Appendix) similar to those reported in recovering secondary forests in Ecuador, 2.9 Mg C ha^-1^ year^-1^ [19]. Within the Yanacocha Andean forest increased C-sequestration rates were generally associated with more mature (large diameter class) trees, i.e. the highest AGB-C sequestration rates were observed in forest plots that already had on average large diameter trees (Fig 5). The RDA result evidenced that higher AGB-C uptake was related to the tree composition of each plot. Those plots with dominance of *Polylepis pauta* and lower prevalence of *Baccharis padifolia* had a positive relationship with higher AGB-C gains. These results suggest the importance large-statured trees, community species composition and their abundances in controlling C of dynamics as previously reported for high tropical and lowlands forests [84,89–92]. Further explanation of these differential sequestration rates may be related to the recent heterogeneous human impact that each plot experienced. We infer that as time passes, and consequently more trees mature, the natural regeneration process in the Andean forest of the Yanacocha Reserve will lead to higher and similar C sequestration rates among plots. Even so, our observation of a positive relationship between trees size and C sequestration rates requires further research combined with a prolonged monitoring period that can provide more insights on the natural recovery dynamics of this ecosystem.

AGB-C increase rates in the Yanacocha páramo, 1.5 Mg C ha^-1^ year^-1^ (see S3 Appendix), are towards the upper end of C sequestration rates reported by other authors, 0.8–1.5 Mg C ha^-1^ year^-1^. Contrary to our third hypothesis, higher AGB-C gains occurred mainly within plots with a relatively high functional diversity and at higher elevations (Fig 7A). A similar relationship of increasing C sequestration with high diversity observed in studies of temperate grasslands [49] might be applicable to the páramo, suggesting the importance of growth-form functional diversity in ecosystem functionality. The correlation between AGB-C sequestration rates and altitude in the Yanacocha páramo might be also related to decreasing human impact with increasing elevation [93]. The gains in AGB-C in the Yanacocha Reserve, are similar to rates observed in moderately degraded grassland ecosystems, suggesting an intermediate stage of recovery of the páramo at higher elevations. The link between higher AGB sequestration rates and decreasing disturbance within the páramo may indicate that natural (passive) regeneration techniques likely represent a very effective strategy for regaining AGB in degraded areas, and that intervention that stimulates successional vegetation dynamics leading to an increase in growth-form diversity could accelerate the process further.

AGN-C stocks’ increases were found for the Andean forest and páramo in the Yanacocha Reserve. By comparing 2012 with 2014 census data the same significant gain rate was recorded for both ecosystems, 1.3 Mg C ha^-1^ year^-1^. AGN-C gains in Yanacocha forest are related to those plots dominated by *Polylepis pauta* and *Escallonia myrtilloides* (Fig 7A). In the páramo of Yanacocha, higher sequestration rates in AGN-C were found in páramo’s plots located at flat terrain sites with low coverage of bryophytes and lichens. Yet, the net positive increment in AGN-C among years, in both ecosystems (2.6 Mg C ha^-1^), may reflect a positive trend towards a less disturbed ecosystem.

#### Belowground biomass C stocks’ recovery

The BGB-C (0–36 cm depth) compartment did not present a clear trend between the two censuses within the Yanacocha Reserve. Increases in BGB-C stock values in the Andean forest (0.65 Mg C ha^-1^ year^-1^) and negative values in the páramo (-1.3 Mg C ha^-1^ year^-1^) were not significant. The rate of BGB-C sequestration observed in the Yanacocha forest is within the range of previous studies [7], albeit these results are the first one to report on fine roots BGB-C dynamics above 3,800 m.a.s.l. in *Polylepis* stands. The ordination result showed a clear trend in which the plots dominated by *Baccharis padifolia* evidenced higher BGB-C increments to the plots dominated by *Polylepis pauta*. This finding suggests different C accumulation rates in the root system among dominant species. Plots dominated by *Baccharis padifolia* suggest an earlier successional stage of the forest to those dominated by *Polylepis pauta* that are indicative of a more mature successional stage of the forest.

Negative differences in BGB-C stocks in the Yanacocha páramo were predominantly in the plots located above 4,200 m.a.s.l. suggesting limitations in our sampling method. Since we did not measure coarse roots (≥ 2 mm diameter), we suspect finest roots (< 2 mm) might be under sampled resulting in an underestimation of BGB pool, particularly in plots dominated by coarse-root growth-forms such as shrubs and basal rosettes. Consequently, additional data are required in order to clarify the factors that drive spatiotemporal trends in BGB-C. Additionally, improving the sampling methodology to include coarse roots as well as include an analysis of short-term turnover in root biomass could help strengthen the sampling protocol, and reduce the uncertainty related to paramo BGB productivity.

## Conclusions

The C stocks across the elevational gradient of the Yanacocha reserve were considerably lower (mean 125 Mg C ha^-1^) than those reported for undisturbed sites in similar ecosystems. The lower C stocks in Yanacocha Reserve are likely the legacy of the cattle grazing, fires and timber extraction that occurred in the reserve 23 years ago. Despite the multi-decadal impact of past human activity, the observed C sequestration rates (2.5 Mg C ha^-1^ year^-1^ and 1.5 Mg C ha^-1^ year^-1^ in forest and páramo, respectively) suggest a gradual recovery of C stocks is underway. Carbon sequestration rates in the Yanacocha reserve were positive in both forest and páramo ecosystems and indicate that the aboveground pools are recovering faster than the belowground pools. In the Andean forest, higher accumulation rates in AGB were found in plots with a prevalence of mature trees, whereas in the páramo grasslands the temporal variations were explained by a combination of elevation and plant functional diversity. Higher gains of páramo AGB-C were observed at higher elevations in plots with high levels of growth-forms diversity.

AGN accumulation rates were found to be increasing in páramo plots located in flat terrain irrespective of their altitudinal position, whereas higher BGB accumulation rates were associated with páramo plots located at lower elevations. C stocks allocation in aboveground and belowground compartments in the high Andean indicates that the preservation of the SOC pool is of paramount importance if ecosystems are to be managed to maximize their potential C stocks. Furthermore, we expect that over the long term the Yanacocha Reserve will keep acting as an aboveground C sink with AGB values reaching similar values as those reported for less degraded Andean ecosystems.

To our knowledge, the results presented here are some of the first to quantify C stocks and their temporal variation using repeated surveys of the same sites in degraded high Andean ecosystems. These findings help to elucidate the natural environmental factors driving the dynamics in high Andean C stocks. The design and implementation of low-cost restoration strategies in Andean forests and páramos may include cost-benefit analyses considering biomass accumulation rates for human assisted and natural restoration processes, taking into account the degree of disturbance, the landscape context and the local environmental variables to maximize the ecological and environmental benefits expected from restoration actions. As our results suggest the use of biomass allocation and productivity as functional indicators may provide insight to the process of assessing ecosystem recovery along environmental and temporal gradients. However, a longer period of continuous monitoring will improve our results and provide a more robust dataset upon which guide restoration actions to contribute to secure environmental services, including climate change mitigation.

## Supporting information

S1 Fig Community structure for each Andean forest plot by diametric class and species frequency for the first (FC) and second (SC) censuses of the Yanacocha Reserve. (DOCX)

S1 Table Number of tree individuals and average trunk diameter for each Andean forest plot for the first (FC) and second (SC) censuses of the Yanacocha Reserve. (DOCX)

S2 Table Average C-stock (± 1 standard deviation) for the first and second censuses (italics) in Mg C ha-1 in the Andean forest and páramo of the Yanacocha Reserve, Pichincha, Ecuador. Cells bordered with thick lines represent non-significant differences in compartments between censuses (95% confidence level and p ≥ 0.05). Shaded cells represent non-significant differences in compartments between habitats (95% confidence level and p ≥ 0.05). In all the cases, a t-test was used. FC: First Census; SC = Second Census.
(DOCX)

S1 Appendix Multispecies allometric equations to estimate Andean forest aboveground biomass. (DOCX)

S2 Appendix (XLSX)

S3 Appendix (XLSX)
