# Supplementary material for: Carbon sequestration rates indicate ecosystem recovery following human disturbance in the equatorial Andes
Source: PLoS One. 2020 Mar 30;15(3):e0230612. doi: 10.1371/journal.pone.0230612 (PMC7105124; doi:10.1371/journal.pone.0230612)
Supplement: S1 Fig — (DOCX) [file pone.0230612.s001.docx]

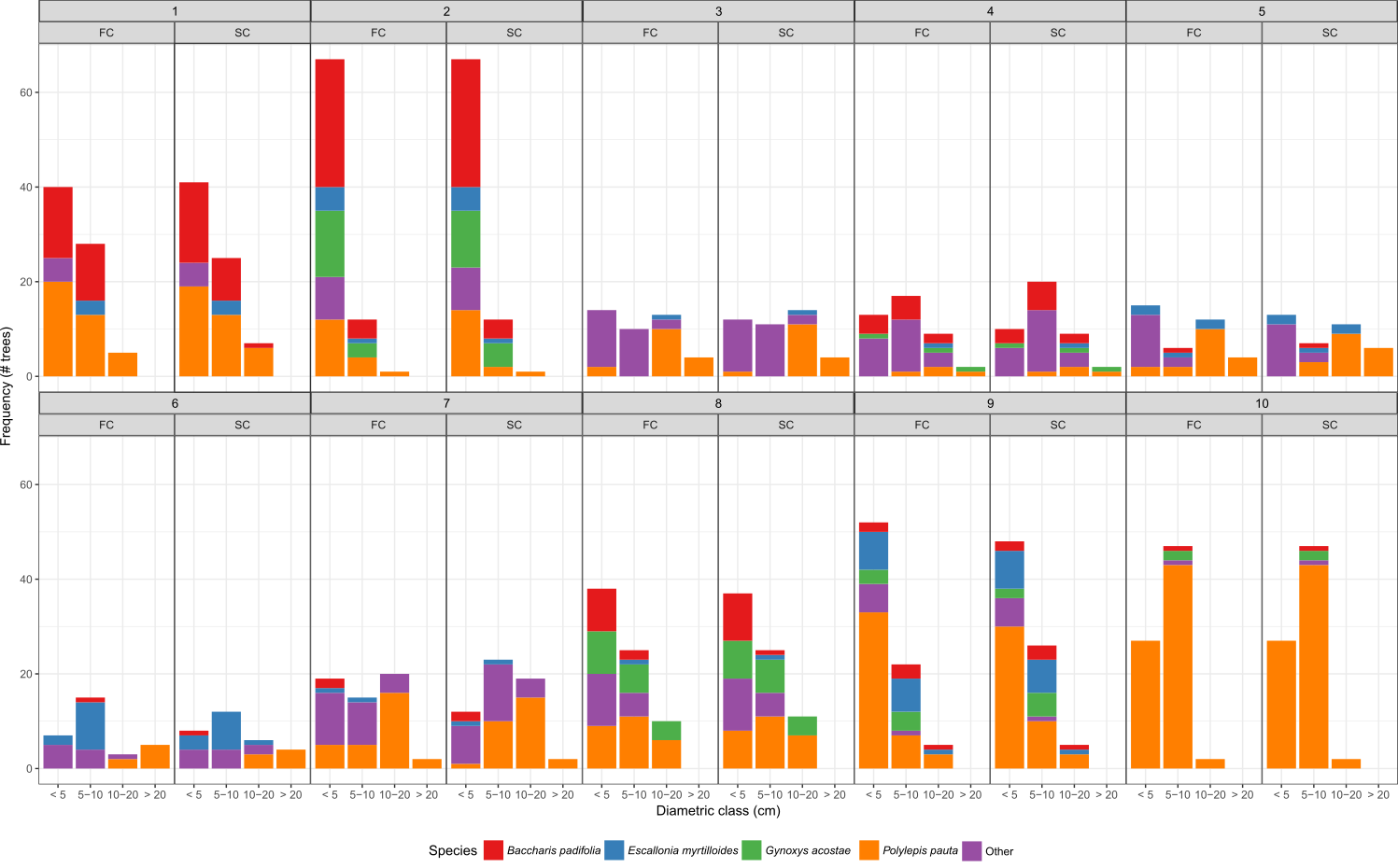


Figure S1: Community structure for each Andean forest plot by diametric class and species frequency for the first (FC) and second (SC) censuses of the Yanacocha Reserve
