# Supplementary material for: Carbon sequestration rates indicate ecosystem recovery following human disturbance in the equatorial Andes
Source: PLoS One. 2020 Mar 30;15(3):e0230612. doi: 10.1371/journal.pone.0230612 (PMC7105124; doi:10.1371/journal.pone.0230612)
Supplement: S1 Table — (DOCX) [file pone.0230612.s002.docx]

Table S1: Number of tree individuals and average trunk diameter for each Andean forest plot for the first (FC) and second (SC) censuses of the Yanacocha Reserve.

|  |  | **Number of**  **individuals** | | **Average trunk**  **diameter (cm) (± 1SD)** | |
| --- | --- | --- | --- | --- | --- |
| **Plot** | **Diametric class** | **FC (2012)** | **SC (2012)** | **FC (2012)** | **SC (2012)** |
| **1** | 2.5-4.9 | 36 | 35 | 3.42 (0.6) | 3.44 (0.6) |
|  | 5-10.9 | 31 | 23 | 6.74 (1.7) | 6.95 (1.5) |
|  | 11-20.9 | 3 | 7 | 13.7 (1.9) | 13.06 (1.8) |
| **Total 1** |  | **70** | **65** | **5.33 (2.7)** | **5.72 (3.2)** |
| **2** | 2.5-4.9 | 71 | 64 | 3.31 (0.6) | 3.42 (0.6) |
|  | 5-10.9 | 12 | 11 | 6.98 (1.2) | 6.97 (1.5) |
|  | 11-20.9 | 1 | 1 | 14 | 15.2 |
| **Total 2** |  | **84** | **76** | **3.96 (1.9)** | **4.09 (2)** |
| **3** | 2.5-4.9 | 12 | 13 | 3.56 (0.6) | 3.43 (0.7) |
|  | 5-10.9 | 11 | 12 | 6.61 (1.5) | 6.6 (1.6) |
|  | 11-20.9 | 13 | 13 | 14.49 (2.7) | 13.91 (2.2) |
|  | >21 | 3 | 4 | 26.4 (2.8) | 25.45 (3.8) |
| **Total 3** |  | **39** | **42** | **9.82 (6.9)** | **9.68 (7)** |
| **4** | 2.5-4.9 | 17 | 9 | 3.89 (0.6) | 3.82 (0.6) |
|  | 5-10.9 | 18 | 20 | 6.93 (1.6) | 6.91 (1.6) |
|  | 11-20.9 | 8 | 8 | 14.76 (3.4) | 15.01 (3.4) |
|  | >21 | 2 | 2 | 22.5 (0.8) | 23 (0.7) |
| **Total 4** |  | **45** | **39** | **7.87 (5.3)** | **8.68 (5.5)** |
| **5** | 2.5-4.9 | 16 | 14 | 3.74 (0.6) | 4.03 (0.7) |
|  | 5-10.9 | 7 | 8 | 8.16 (1.5) | 8.45 (1.5) |
|  | 11-20.9 | 11 | 12 | 14.5 (2.8) | 15.16 (3.3) |
|  | >21 | 4 | 4 | 24.85 (2.2) | 25.38 (2.3) |
| **Total 5** |  | **38** | **38** | **9.89 (7.1)** | **10.72 (7.2)** |
| **6** | 2.5-4.9 | 9 | 7 | 3.48 (0.7) | 3.77 (0.8) |
|  | 5-10.9 | 16 | 14 | 6.94 (1.7) | 7.34 (1.8) |
|  | 11-20.9 | 3 | 3 | 16.5 (4.5) | 16.47 (4) |
|  | >21 | 4 | 4 | 25.95 (5.9) | 26.26 (6.2) |
| **Total 6** |  | **32** | **28** | **9.24 (7.7)** | **10.13 (8)** |
| **7** | 2.5-4.9 | 23 | 11 | 3.81 (0.7) | 3.75 (0.8) |
|  | 5-10.9 | 18 | 21 | 7.49 (1.9) | 7.35 (1.8) |
|  | 11-20.9 | 17 | 17 | 15.14 (2.1) | 15.84 (2.3) |
|  | >21 | 2 | 2 | 24.65 (1.8) | 25.28 (1.9) |
| **Total 7** |  | **60** | **51** | **8.82 (5.7)** | **10.11 (6)** |
| **8** | 2.5-4.9 | 49 | 28 | 3.57 (0.6) | 3.78 (0.7) |
|  | 5-10.9 | 27 | 24 | 7.27 (1.7) | 7.47 (1.7) |
|  | 11-20.9 | 8 | 10 | 12.34 (1.5) | 12.57 (1.8) |
| **Total 8** |  | **84** | **62** | **5.6 (3)** | **6.63 (3.4)** |
| **9** | 2.5-4.9 | 49 | 48 | 3.42 (0.6) | 3.6 (0.7) |
|  | 5-10.9 | 22 | 25 | 6.8 (1.3) | 7.06 (1.3) |
|  | 11-20.9 | 5 | 5 | 13.56 (2.6) | 13.9 (2.7) |
| **Total 9** |  | **76** | **78** | **5.06 (2.9)** | **5.37 (3)** |
| **10** | 2.5-4.9 | 47 | 47 | 3.55 (0.7) | 3.54 (0.6) |
|  | 5-10.9 | 52 | 51 | 6.56 (1.2) | 6.81 (1.3) |
|  | 11-20.9 | 3 | 4 | 13.7 (3.3) | 13.66 (3.3) |
| **Total 10** |  | **102** | **102** | **5.38 (2.4)** | **5.57 (2.6)** |
| **Total among plots** | | **630** | **581** | **6.42 (4.7)** | **6.94 (5)** |
