# Supplementary material for: Carbon sequestration rates indicate ecosystem recovery following human disturbance in the equatorial Andes
Source: PLoS One. 2020 Mar 30;15(3):e0230612. doi: 10.1371/journal.pone.0230612 (PMC7105124; doi:10.1371/journal.pone.0230612)
Supplement: S2 Table — Cells bordered with thick lines represent non-significant differences in compartments between censuses (95% confidence level and p ≥ 0.05). Shaded cells represent non-significant differences in compartments between habitats (95% confidence level and p ≥ 0.05). In all the cases, a t-test was used. FC: First Census; SC = Second Census. (DOCX) [file pone.0230612.s003.docx]

Table S2: Average C-stock (± 1 standard deviation) for the first and second censuses (italics) in Mg C ha^-1^ in the Andean forest and páramo of the Yanacocha Reserve, Pichincha, Ecuador. Cells bordered with thick lines represent non-significant differences in compartments between censuses (95% confidence level and p ≥ 0.05). Shaded cells represent non-significant differences in compartments between habitats (95% confidence level and p ≥ 0.05). In all the cases, a t-test was used. FC: First Census; SC= Second Census.

|  | **Andean forest (n=10)** | | | **páramo (n=29)** | | |
| --- | --- | --- | --- | --- | --- | --- |
|  | FC (2012) | *SC (2014)* | Absolute / relative variation | FC (2012) | *SC (2014)* | Absolute / relative variation |
| **AGB** | 33.7 (±22.2) | *38.6 (±23.7)* | 4.9/31.8 | 4.2 (±1.8) | *7.2 (±5.1)* | 3/65.3 |
| **AGN** | 4.2 (±1.5) | *6.8 (±3.3)* | 2.6/83.9 | 7.3 (±3.7) | *9.9 (±4.8)* | 2.5/202 |
| **BGB** | 10.7 (±4.3) | *12 (± 5.7)* | 1.3/29.2 | 13.8 (±7.7) | *11.2 (±6.1)* | -2.6/-1.7 |
| **SOC**  **0-18 cm** | 37.9 (±18.1) | *-* | - | 70.3 (±17.6) | *-* | *-* |
| **SOC**  **18-36 cm** | 25.5 (±11.4) | *-* | - | 43.1 (±17.6) | *-* | *-* |
| **TCC** | 112 (±26.8) | - | - | 138.7 (±32.8) | *-* | *-* |
