# Supplementary material for: Carbon sequestration rates indicate ecosystem recovery following human disturbance in the equatorial Andes
Source: PLoS One. 2020 Mar 30;15(3):e0230612. doi: 10.1371/journal.pone.0230612 (PMC7105124; doi:10.1371/journal.pone.0230612)
Supplement: S1 Appendix — (DOCX) [file pone.0230612.s004.docx]

**Appendix S1**

**Multispecies allometric equations to estimate Andean forest Aboveground Biomass**

Two nonlinear regression models produced the best estimations of aboveground biomass (AGB) in the recovering Andean forest of the Yanacocha Reserve: one using only the trunk diameter (cm) (Equation 1, Table I) and one using the trunk diameter and the tree height (cm) (Equation 2, Table I) as predictors variables. Table II contains the raw data of the harvested trees used for developing the models.

**Table I:** Allometric equations developed for estimating the AGB in the recovering Andean forest in the Yanacocha Reserve. a and b are the coefficients of the equations; AIC: Akaike Information Criterion; BIC: Bayesian Information Criterion; RMSE: Root Mean Squared Error. AGB: aboveground biomass (kg), DBH: trunk diameter at breast height (cm), H: tree height (cm). The number of degrees of freedom for both models was 34.

|  |  | Coefficients | |  |  |  |  |
| --- | --- | --- | --- | --- | --- | --- | --- |
|  | **Allometric models** | **a** | **b** | **R^2^** | **AIC** | **BIC** | **RMSE** |
| Equation 1 | $\text{AGB}\text{=a}{\text{∙D}\text{BH}}^{\text{b}}$ | 0.041 | 2.56 | 0.919 | 264.96 | 268.13 | 9.33 |
| Equation 2 | $\text{AGB}\text{=}\text{a∙H}\text{∙}{\text{D}\text{BH}}^{\text{b}}$ | 1.94x10^-4^ | 2.126 | 0.961 | 238.44 | 241.6 | 6.46 |

The regression coefficients (R^2^) and the AIC and BIC criteria indicate that the developed models offer a strong performance for predicting AGB. Although both models presented a positive bias (5.39% for model including tree height and 6.60% with no tree height) no clear trend was present in terms of biomass overestimation. In contrast with other multispecies allometric models [1, 2], wood density did not improve the performance of the allometric models. The correlation between AGB and wood density was rather low (R^2^ = 0.265). The same trend was found when wood density was compared to individual trunk diameters for all species, suggesting that wood density it is not related neither with the trunk diameter nor with aboveground biomass in this site. These results are in concordance with the findings presented by Stegen, Swenson [3], the authors found no consistent correlation between individual wood density and individual AGB (expressed as basal area) among the tropical forests sampled. Picard, Saint-André [4] remark that wood density may be similar in trees from forests growing in similar conditions (including location, access, clearing and slope) being representative of the local environment.

The model including tree height reported better regression metrics than the model developed using only the tree diameter. Similar behavior has been reported by different authors for lowland tropical forests [1, 2, 5-7] and for some tropical montane forests [8]. The positive effects of including tree height as predictor variable on the performance of allometric models has been reported extensively [1, 2, 6, 9]; however, for this site its inclusion in the allometric model might be problematic due to field limitations when measuring this variable in high Andean forests. We found inconsistencies in tree height measurements between censuses that might be related with the architecture of the trees as they differ from lowland forest, being more ramified and sinuous [10]. Chave, Condit [11] noted that errors in height measurement lead to errors in estimating AGB. They also pointed out that these errors are propagated to the AGB estimates trough the allometric model by increasing the model function *f* in Taylor series. In this study, to reduce the intrinsic errors in the field estimation of tree height and the total uncertainty of the models, the model including only trunk diameter as predictor variable was used for determining the forest AGB in both censuses.

**References in the supplemental material**

1. Chave J, Réjou‐Méchain M, Búrquez A, Chidumayo E, Colgan MS, Delitti WB, et al. Improved allometric models to estimate the aboveground biomass of tropical trees. Global change biology. 2014;20(10):3177-90.

2. Alvarez E, Duque A, Saldarriaga J, Cabrera K, de las Salas G, del Valle I, et al. Tree above-ground biomass allometries for carbon stocks estimation in the natural forests of Colombia. Forest Ecology and Management. 2012;267:297-308. doi: 10.1016/j.foreco.2011.12.013.

3. Stegen JC, Swenson NG, Valencia R, Enquist BJ, Thompson J. Above‐ground forest biomass is not consistently related to wood density in tropical forests. Global ecology and biogeography. 2009;18(5):617-25.

4. Picard N, Saint-André L, Henry M. Manual for building tree volume and biomass allometric equations: from field measurement to prediction. Food and Agricultural Organization of the United Nations, 2012.

5. Chave J, Andalo C, Brown S, Cairns M, Chambers J, Eamus D, et al. Tree allometry and improved estimation of carbon stocks and balance in tropical forests. Oecologia. 2005;145(1):87-99.

6. Feldpausch TR, Lloyd J, Lewis SL, Brienen R, Gloor E, Mendoza AM, et al. Tree height integrated into pan-tropical forest biomass estimates. Biogeosciences Discussions. 2012;9(3).

7. Feldpausch TR, Banin L, Phillips OL, Baker TR, Lewis SL, Quesada CA, et al. Height-diameter allometry of tropical forest trees. 2011.

8. Vásquez E, Ladd B, Borchard N. Carbon storage in a high-altitude Polylepis woodland in the Peruvian Andes. Alpine botany. 2014;124(1):71-5.

9. Brown S. Estimating biomass and biomass change of tropical forests: a primer: Food & Agriculture Org.; 1997.

10. Clark DA, Brown S, Kicklighter DW, Chambers JQ, Thomlinson JR, Ni J. Measuring net primary production in forests: concepts and field methods. Ecological Applications. 2001;11(2):356-70.

11. Chave J, Condit R, Aguilar S, Hernandez A, Lao S, Perez R. Error propagation and scaling for tropical forest biomass estimates. Philosophical Transactions of the Royal Society of London Series B: Biological Sciences. 2004;359(1443):409-20.

Table II: Harvested tree used for developing the biomass allometric equations

| **Nº Tree** | **Species** | **DBH (cm)** | **Height (cm)** | **Wood density (g/cm^3^)** | **Dry biomass (g)** |
| --- | --- | --- | --- | --- | --- |
| **1** | *Polylepis pauta* | 7.1 | 395 | 0.433 | 6031.4 |
| **2** | *Polylepis pauta* | 6.7 | 548 | 0.428 | 8557.6 |
| **3** | *Polylepis pauta* | 8.7 | 545 | 0.454 | 15911.9 |
| **4** | *Polylepis pauta* | 9.6 | 478 | 0.425 | 19968.0 |
| **5** | *Polylepis pauta* | 15.3 | 909 | 0.797 | 75453.4 |
| **6** | *Polylepis pauta* | 8.9 | 733 | 0.503 | 13448.5 |
| **7** | *Polylepis pauta* | 6.4 | 404 | 0.51 | 5415.1 |
| **8** | *Polylepis pauta* | 10.5 | 879 | 0.479 | 16328.8 |
| **9** | *Polylepis pauta* | 8.4 | 630 | 0.555 | 5240.3 |
| **10** | *Polylepis pauta* | 5.6 | 605 | 0.481 | 2370.1 |
| **11** | *Polylepis pauta* | 7.7 | 503 | 0.451 | 3281.8 |
| **12** | *Polylepis pauta* | 13.4 | 766 | 0.504 | 19058.1 |
| **13** | *Polylepis pauta* | 5.5 | 636 | 0.525 | 5338.2 |
| **14** | *Polylepis pauta* | 7.1 | 601 | 0.416 | 9929.0 |
| **15** | *Polylepis pauta* | 12.5 | 583 | 0.421 | 21033.0 |
| **16** | *Polylepis pauta* | 11.2 | 616 | 0.628 | 23163.7 |
| **17** | *Polylepis pauta* | 10 | 598 | 0.595 | 11188.6 |
| **18** | *Polylepis pauta* | 22.3 | 1075 | 0.702 | 144271.3 |
| **19** | *Polylepis pauta* | 14.4 | 630 | 0.467 | 46789.1 |
| **20** | *Polylepis pauta* | 23.3 | 689 | 0.5 | 118720.5 |
| **21** | *Polylepis pauta* | 16 | 567 | 0.724 | 45924.4 |
| **22** | *Escallonia myrtilloides* | 11.7 | 500 | 0.438 | 18239.1 |
| **23** | *Escallonia myrtilloides* | 8 | 427 | 0.474 | 6196.2 |
| **24** | *Escallonia myrtilloides* | 6.5 | 359 | 0.442 | 5012.5 |
| **25** | *Escallonia myrtilloides* | 12.9 | 539 | 0.592 | 17196.3 |
| **26** | *Escallonia myrtilloides* | 7.5 | 518 | 0.443 | 8259.3 |
| **27** | *Baccharis padifolia* | 10.3 | 570 | 0.59 | 13139.1 |
| **28** | *Baccharis padifolia* | 8.2 | 365 | 0.604 | 7803.7 |
| **29** | *Baccharis padifolia* | 6.1 | 484 | 0.584 | 5388.4 |
| **30** | *Baccharis padifolia* | 6.2 | 466 | 0.578 | 4161.3 |
| **31** | *Gynoxys acostae* | 6.2 | 405 | 0.555 | 4918.4 |
| **32** | *Gynoxys acostae* | 7.4 | 390 | 0.471 | 10571.6 |
| **33** | *Gynoxys acostae* | 8 | 402 | 0.816 | 9197.7 |
| **34** | *Gynoxys acostae* | 10.4 | 663 | 0.796 | 16211.8 |
| **35** | *Gynoxys acostae* | 15 | 537 | 0.674 | 33953.0 |
| **36** | *Gynoxys acostae* | 21.2 | 690 | 0.448 | 82577.2 |
